# Human Dental Pulp Stem Cells and Gingival Fibroblasts Seeded into Silk Fibroin Scaffolds Have the Same Ability in Attracting Vessels

**DOI:** 10.3389/fphys.2016.00140

**Published:** 2016-04-19

**Authors:** Anna Woloszyk, Johanna Buschmann, Conny Waschkies, Bernd Stadlinger, Thimios A. Mitsiadis

**Affiliations:** ^1^Orofacial Development and Regeneration, Center of Dental Medicine, Institute of Oral Biology, University of ZurichZurich, Switzerland; ^2^Plastic Surgery and Hand Surgery, University Hospital ZurichZurich, Switzerland; ^3^Institute for Biomedical Engineering, ETH and University of ZurichZurich, Switzerland; ^4^Visceral and Transplant Surgery, University Hospital ZurichZurich, Switzerland; ^5^Clinic of Cranio-Maxillofacial and Oral Surgery, University of Zurich, University Hospital ZurichZurich, Switzerland

**Keywords:** regenerative medicine, vascularization, mesenchymal stem cells, human gingival fibroblasts (hGFs), human dental pulp stem cells (hDPSCs), silk fibroin scaffolds, chorioallantoic membrane (CAM)

## Abstract

Neovascularization is one of the most important processes during tissue repair and regeneration. Current healing approaches based on the use of biomaterials combined with stem cells in critical-size bone defects fail due to the insufficient implant vascularization and integration into the host tissues. Therefore, here we studied the attraction, ingrowth, and distribution of blood vessels from the chicken embryo chorioallantoic membrane into implanted silk fibroin scaffolds seeded with either human dental pulp stem cells or human gingival fibroblasts. Perfusion capacity was evaluated by non-invasive *in vivo* Magnetic Resonance Imaging while the number and density of blood vessels were measured by histomorphometry. Our results demonstrate that human dental pulp stem cells and gingival fibroblasts possess equal abilities in attracting vessels within silk fibroin scaffolds. Additionally, the prolonged *in vitro* pre-incubation period of these two cell populations favors the homogeneous distribution of vessels within silk fibroin scaffolds, which further improves implant survival and guarantees successful healing and regeneration.

## Introduction

Classical bone defect treatments require the use of anatomically adapted devices that can establish tissue functionality and provide relief of symptoms to patients. However, the effectiveness and durability of treatments involving orthopedic or maxillofacial implants and transplantations of autologous bone grafts are still debatable. Indeed, the outcome of these regenerative solutions may be compromised by a variety of iatrogenic complications such as tissue morbidity and/or inflammation following implant or graft transplantation. Stem cell-based therapeutic approaches offer attractive alternatives in clinics since they can promise physiologically improved structural and functional outcomes. These therapies require a significant number of stem cell populations for implantation into specifically designed and composed scaffolds with various biological activities and compositions that can ensure their fast integration into the defect site. To avoid post-operative complications it is essential to promote rapid, constant, and complete vascularization of these implantable constructs (Giannicola et al., 2010). Different strategies have been developed in order to enhance the vascularization capabilities of the various implanted materials. Cell seeding is one of the most popular and beneficial strategies to achieve this important goal. For example, it has been demonstrated that bone marrow stem cells (BMSCs) and endothelial cells seeded together in decalcified bone scaffolds can accelerate the vascularization process during calvaria bone repair (Koob et al., 2011). Similarly, it has been shown that human amniotic fluid-derived stem cells are able to enhance and stabilize vessel attraction and formation within collagen-chondroitin sulfate scaffolds (Lloyd-Griffith et al., 2015). Cell-seeded grafts may also have immunosuppressive functions that allow an improved healing procedure, as it has been already shown for adipose-derived stem cell-seeded scaffolds (Plock et al., 2015).

The use of human dental pulp stem cells (hDPSCs; Gronthos et al., 2000; Shi et al., 2001; Huang et al., 2009) and human gingival fibroblasts (hGFs; Xu et al., 2013; Chiquet et al., 2015) for regenerative purposes has been proposed as an alternative to BMSCs, since these cell populations exhibit similar properties and have the ability to adopt a variety of alternative fates in response to extrinsic factors. Indeed, it has been shown that hGFs have distinct functional activities in the regeneration and repair of periodontal tissues (Lee et al., 2013; Chiquet et al., 2015). Similarly, numerous studies have demonstrated that hDPSCs have the potential to differentiate into different cell types such as myocytes, chondrocytes, adipocytes, neurons, and osteoblasts both *in vitro* and *in vivo* (Gronthos et al., 2000; Zhang et al., 2006; d'Aquino et al., 2007; Bluteau et al., 2008; Mitsiadis et al., 2015). In addition, *in vitro* and *in vivo* studies have shown that hDPSCs may affect endothelial cell behavior by enhancing their migration and attraction toward them (Hilkens et al., 2014). The first clinical trial using autologous hDPSCs combined with commercially available collagen scaffolds (i.e., Gingistat®) for alveolar bone reconstruction has been successfully performed several years ago (d'Aquino et al., 2009). A 3 years follow-up study has shown that the structure of the regenerated bone at the grafted site was more compact than normal spongy alveolar bone (Giuliani et al., 2013), thus indicating that the choice of the appropriate scaffold and/or stem cell population is crucial for targeted, tissue-specific, regenerative procedures.

Silk fibroin scaffolds are commonly used in the medical field for a diverse set of applications such as vascular, neuronal, skin, cartilage, and bone regeneration (Altman et al., 2003; Kundu et al., 2013). Using bioreactor devices, we have previously shown that hDPSC-seeded silk fibroin scaffolds are able to form mineralized structures *in vitro* in a very short period of time (Woloszyk et al., 2014). In another recent study using the chicken embryo chorioallantoic membrane (CAM) assay combined with magnetic resonance imaging (MRI), we have demonstrated that hDPSCs can attract vessels within silk fibroin scaffolds (Kivrak Pfiffner et al., 2015).

Here we extended our previous studies and compared the capacity of hDPSCs and human gingival fibroblasts (hGFs) to attract vessels within silk fibroin scaffolds. Vascularization of the silk fibroin scaffolds was assessed using MRI and histomorphometric measurements. The results clearly demonstrated that hDPSCs and hGFs have similar abilities in attracting vessels and thus could be equally used in clinics for generating richly vascularized tissues.

## Materials and methods

### Production of silk fibroin scaffolds

Silk fibroin scaffolds were produced using the salt leaching technique as previously described (Sofia et al., 2001; Nazarov et al., 2004; Hofmann et al., 2007). Briefly, silkworm cocoons (Trudel Inc., Zurich, Switzerland) were boiled in 0.02 M sodium carbonate (Fluka AG, Buchs SG, Switzerland) and rinsed with ultrapure water (UPW) to extract sericin. After drying, the silk was dissolved in 9 M lithium bromide and dialyzed against UPW for 36 h followed by lyophilization (Alpha 1-2, Martin Christ GMBH, Osterode am Harz, Germany). A 17% (w/v) silk fibroin solution was prepared by dissolving lyophilized silk in 1,1,1,3,3,3-hexafluoro-2-propanol (HFIP) (abcr GmbH & Co., Karlsruhe, Germany). This solution was added to Teflon containers filled with sodium chloride (Sigma-Aldrich Chemie GmbH, Buchs SG, Switzerland) at a ratio of 1:20 (silk fibroin:NaCl). After the evaporation of HFIP, the blocks were immersed in 90% methanol for 30 min (Sofia et al., 2001). The scaffolds were dried for at least 48 h before sodium chloride was leached out in five changes of UPW in 48 h resulting in scaffolds with more than 90% porosity (Nazarov et al., 2004). Wet silk fibroin scaffolds were cut into cylinders of 5 mm diameter and 3 mm height (59 mm^3^) and were sterilized by autoclaving at 121°C and 1 bar for 20 min.

### Cell culture

The procedure for anonymized cell collection was approved by the Kantonale Ethikkommission of Zurich (reference number 2012-0588) and performed with written patients' consent. Human dental pulp stem cells (hDPSCs) were isolated from the dental pulp of extracted impacted wisdom teeth of healthy patients as previously described (Tirino et al., 2012). The dental pulps were enzymatically digested for 1 h at 37°C in a solution of collagenase (3 mg/mL; Life Technologies Europe B.V., Zug ZG, Switzerland) and dispase (4 mg/mL; Sigma-Aldrich Chemie GmbH, Buchs SG, Switzerland). A filtered single-cell suspension was plated in a 40 mm Petri dish with hDPSC growth medium containing DMEM/F12 (Sigma-Aldrich Chemie GmbH, Buchs SG, Switzerland) with 10% fetal bovine serum (FBS) (PAN Biotech GmbH, Aidenbach, Germany), 1% penicillin/streptomycin (P/S) (Sigma-Aldrich Chemie GmbH, Buchs SG, Switzerland), 1% L-glutamine (Sigma-Aldrich Chemie GmbH, Buchs SG, Switzerland), and 0.5 μg/ml fungizone (Life Technologies Europe B.V., Zug ZG, Switzerland) after washing away the enzyme solution. Healthy parts of gingiva were collected from biopsies. Gingival tissues were washed in phosphate buffered saline (PBS) (Life Technologies Europe B.V., Zug ZG, Switzerland), sectioned into small pieces and placed in 35 mm Petri dishes (TPP Techno Plastic Products AG, Trasadingen SH, Switzerland) for the outgrowth of human gingival fibroblasts (hGFs). The fibroblast growth medium is composed by high glucose DMEM (Life Technologies Europe B.V., Zug ZG, Switzerland), 10% FBS, 1% P/S, and 1% HEPES (Sigma-Aldrich Chemie GmbH, Buchs SG, Switzerland). hDPSCs were cultured in DMEM/F12 (Sigma-Aldrich Chemie GmbH, Buchs SG, Switzerland) supplemented with 10% fetal bovine serum (FBS) (Biochrom AG, Berlin, Germany), 1% Penicillin/Streptamycin (P/S) (Sigma-Aldrich Chemie GmbH, Buchs SG, Switzerland), and 0.5 μg/mL fungizone (Thermo Fisher Scientific AG, Reinach BL, Switzerland). hGFs were expanded in DMEM high glucose (Thermo Fisher Scientific AG, Reinach BL, Switzerland) supplemented with 10% FBS, 1% P/S, and 1% HEPES (Thermo Fisher Scientific AG, Reinach BL, Switzerland). Cells were passaged at 80–90% confluence. All experiments were performed with cells from passages 4 and 5.

### Scaffold seeding

Sterile silk fibroin scaffolds were seeded with cells at a density of 0.5 × 10^6^ per scaffold and then placed in a humidified incubator for 1 h at 37°C and 5% CO_2_. The cells that were not attached to the scaffolds were washed away and then the seeded scaffolds were incubated for 1 week at 37°C before being placed on the CAM of fertilized chicken eggs. Empty scaffolds were used as controls (data not shown).

### CAM assay

**Figure 1:**
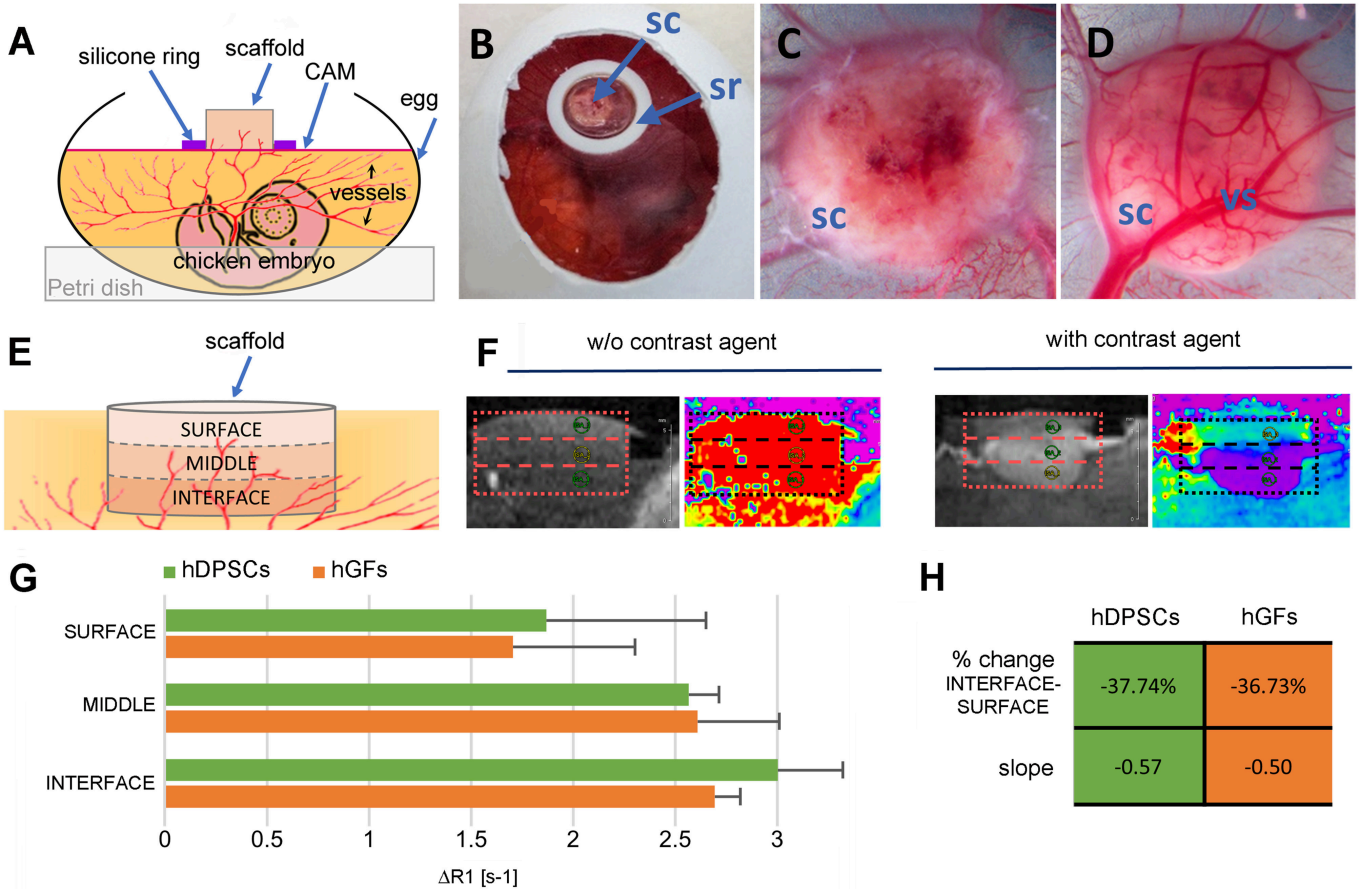
**Experimental setup and MRI analysis. (A,B)** The scaffold is placed in the middle of a silicone ring on top of the CAM of a fertilized egg, which is stabilized in a Petri dish and incubated for 1 week. **(C)** Top and **(D)** bottom views of the scaffold after 1 week of incubation *in ovo*. **(E)** Scheme showing the analyzed parts of the samples. **(F)** Magnetic resonance image of the vascularized scaffold before and after the injection of the contrast agent Gd-DOTA. **(G)** Relaxation rate change ΔR1 in the interface, middle, and surface region of the scaffolds. Values are given as mean ± standard deviation. No statistically significant differences were found. **(H)** Percent change of the mean ΔR1 and slope of regression line between the interface and the surface layers within each group. CAM, chorioallantoic membrane; sc, scaffold; sr, silicone ring; vs, vessel; hDPSCs, human dental pulp stem cells; hGFs, human gingival fibroblasts.

No IACUC approval is necessary when performing experiments in chicken embryos until embryonic day 14 according to Swiss animal care guidelines (TSchV, Art. 112). Fertilized Lohman white LSL chicken eggs (Animalco AG, Staufen AG, Switzerland) were pre-incubated for 3 days at 38°C at a rotation speed of 360°/4 h (Bruja 3000, Brutmaschinen-Janeschitz GmbH, Hammelburg, Germany). On embryonic day 3 (ED 3) the eggs were processed for *in ovo* cultivation, which requires the opening of the shell with a drill (Dremel§, Conrad Electronic AG, Wollerau SZ, Switzerland). 2 mL of albumen was always removed with a syringe to increase the empty space under the top of the egg shell. The eggs were stabilized in 60 mm Petri dishes (Greiner Bio-One GmbH, Frickenhausen, Germany) and the created holes of the shells were covered with another 60 mm Petri dish that was fixed with a tape before the incubation of eggs at 37°C. On ED 7, empty and cell-seeded scaffolds were placed on the CAM (1-2/egg) in the middle of silicone rings that ensure a flat surface (Figures 1A,B) during their incubation period of 7 days.

### *In vivo* assessment of perfusion capacity using MRI

Vascularization of the scaffolds by capillaries of the chicken embryo's CAM was studied on ED 14 using Magnetic Resonance Imaging (MRI) as previously described (Kivrak Pfiffner et al., 2015). The eggs were placed onto a custom-built sliding bed and enveloped by warm water tubing to maintain the temperature of the chicken embryo in a physiological range. To prevent motion, the chicken embryo was sedated with 5 drops of 1:100 M ketamine (Ketasol-100, Dr. E. Graeub AG, Bern BE, Switzerland) dripped onto the CAM surface. MRI was performed with a 4.7 T/16 cm Bruker PharmaScan small animal scanner (Bruker BioSpin MRI GmbH, Ettlingen, Germany) equipped with an actively decoupled two-coil system consisting of a 72 mm bird cage resonator for excitation and a 20 mm single loop surface coil for reception. Anatomical reference images were obtained in coronal, transversal, and sagittal slice orientations. T1-weighted MR images were acquired with a RARE sequence of variable TR and TE for quantitative T1 and T2 mapping. T1 maps were acquired in the samples before and after intravenous injection of 100 μL of 0.05 M Gd-DOTA MRI contrast agent (Dotarem®, Guerbet AG, Zuerich ZH, Switzerland). The time between Gd-DOTA injection and T1 mapping was kept constant at 25 min. T1 relaxation times were determined in three regions of interest: at the interface of the scaffold with the CAM (i.e., lower part), in the middle part of the scaffold, and finally at the surface of the scaffold (i.e., upper part). Perfusion capacity in these three regions was assessed through changes in the longitudinal relaxation rate ΔR1 before and after injection of Gd-DOTA, as the relaxation rate changes with the amount of gadolinium present in the CAM.

### Histological analysis

The scaffold-CAM complex was fixed in 4% paraformaldehyde (PFA) at 4°C overnight. The cell-seeded constructs were excised from the CAM, embedded in paraffin (Haslab GmbH, Ostermundigen BE, Switzerland) and sectioned vertically in 5 μm thick sections. For histological evaluation, sections from the center of the scaffolds were stained with Hematoxylin and Eosin (H&E) (Mayer's hemalum solution, Merck KGaA, Darmstadt, Germany; Eosin Y, Sigma-Aldrich Chemie GmbH, Buchs SG, Switzerland). Pictures were taken using the Axio Scan.Z1 slidescanner (Carl Zeiss AG, Oberkochen, Germany), a Hitachi HV-F202FCL camera (Hitachi, Ltd., Tokyo, Japan), and the ZEN 2012 SP2 software (Carl Zeiss AG, Oberkochen, Deutschland) provided by the Center for Microscopy and Image analysis, University of Zurich.

### Manual vessel analysis

The number of vessels and the percentage of vessels per scaffold area were determined by counting the number of vessels in longitudinal sections taken from the center of the scaffold (i.e., middle part). Each vessel was marked in black and ImageJ v1.48s (National Institutes of Health, USA) was used to analyze the number of marked vessels and their total area. Three sections of each sample (3 × hDPSCs, 3 × hGFs) were analyzed.

### Statistics

For a comparison of the groups a one-way analysis of variance (ANOVA) was performed using GraphPad Prism v6.05 (GraphPad Software, Inc., La Jolla, CA, USA). A Bonferroni's multiple comparisons test was conducted to determine significant differences between the groups. Data were considered significant at *p* < 0.05 (^*^) and highly significant at *p* < 0.01 (^**^).

## Results

### Macroscopic analysis

In growing vessels were visible on the surfaces of the hDPSC- and hGF-seeded silk fibroin scaffolds, both on the top (Figure 1C) and on the bottom (Figure 1D) of the samples after their CAM incubation for 1 week.

### MRI analysis

For a more precise analysis of the perfusion capacity of the samples, the scaffolds were divided into three equally sized regions, specified as “interface” (i.e., bottom of the scaffold touching the CAM), “middle,” and “surface” (Figure 1E). Thereafter, their perfusion capacity was determined by calculating the change of R_1_ relaxation rates (ΔR_1_) measured by MRI before and after contrast enhancement by injected Gd-DOTA, a paramagnetic contrast agent (Figure 1F). The ΔR_1_ values within the three regions of the scaffolds showed a gradual decrease of their perfusion capacity toward the surface region (Figure 1G). A comparison of the perfusion capacity between the hDPSC- and hGF-seeded silk fibroin scaffolds showed no significant differences of ΔR_1_within any of the three scaffold regions (Figure 1G). Both cell-seeded scaffolds showed only a percent change from interface to surface of approximately −37% (Figure 1H) resulting in much flatter slopes of −0.57 (hDPSCs) and −0.50 (hGFs) when compared to a slope of −1.33 observed in empty silk scaffolds (data not shown; Kivrak Pfiffner et al., 2015).

### Histological and manual vessel analysis

**Figure 2:**
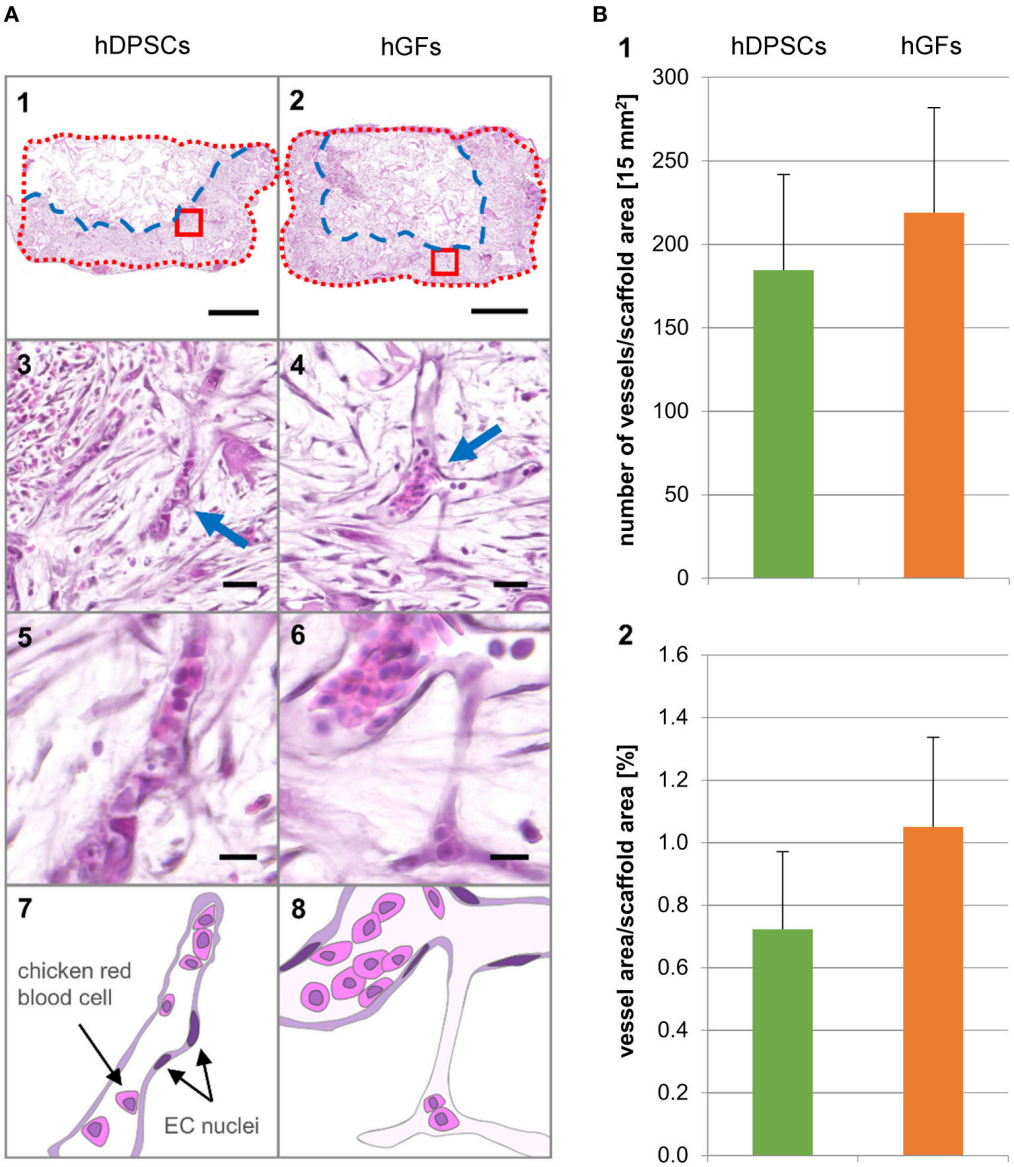
**Hematoxylin and Eosin stainings of longitudinal sections and histomorphometric analysis**. **(A_1, 3, 5, 7_)** hDPSCs-seeded scaffolds. **(A_2, 4, 6, 8_)** hGFs-seeded scaffolds. **(A_1, 2_)** Scaffold section overview. Blue dashed line indicates the front line of the growing tissue. Red dotted line indicates the outline of the scaffold. Scale bar = 1 mm. **(A_3, 4_)** Magnifications showing vascularization in the area marked with a red box in the corresponding overview picture. Arrows indicate capillaries. Scale bar = 25 μm. **(A_5, 6_)** Magnifications showing single capillaries. Scale bar = 10 μm. **(A_7, 8_)** Schematic representation of the capillaries shown in 5 and 6. **(B_1_)** Number of vessels per scaffold area. **(B_2_)** Percent vessel area per scaffold area. Values are given as mean ± standard deviation. No statistically significant differences were found. EC, endothelial cells; hDPSCs, human dental pulp stem cells; hGFs, human gingival fibroblasts.

Qualitative and quantitative analyses of the vascularized scaffolds were performed using sections stained with Hematoxylin and Eosin (H&E). These histological sections showed large areas of tissue expansion (depicted by the blue dashed line) into the scaffold (depicted by the red dotted line) (Figure 2A). While in the samples seeded with hDPSCs the growing tissue filled approximately half of the scaffold areas (Figure 2A1), the tissue occupied roughly two-thirds of the scaffolds seeded with hGFs (Figure 2A2). CAM-derived blood vessels have penetrated both hDPSC- and hGF-seeded scaffolds (Figures 2A3–6). A manual analysis was performed in order to determine the number and the percent area occupied by the vessels within the three defined scaffold areas (Figure 2B). The corresponding graphs showed proportional values for scaffolds seeded with hDPSCs and hGFs. Although the average size of the counted vessels in both scaffolds was the same, a higher number of vessels was counted in hGFs-seeded scaffolds. This is due to the larger area occupied by the tissue in hGFs-seeded scaffolds when compared to the occupied tissue area in hDPSC-seeded scaffolds. The distribution of the vasculature along the horizontal axis of the scaffold was nearly homogeneous, whereas the vessel density decreased along the vertical axis following an interface-middle-surface gradient.

## Discussion

The use of biomaterials combined with stem cells aims at the successful healing and regeneration of injured or pathological tissues and organs (Stock and Vacanti, 2001; Griffith and Naughton, 2002). An essential prerequisite for effective tissue repair is the integration of the grafted material into the host tissue and its fast and effective vascularization, which ensures the constant supply of nutrients and oxygen, thus preventing necrosis of the newly formed tissue (Novosel et al., 2011). Therefore, it is essential and vital in stem cell-based regenerative treatments to immediately attract blood vessels into the implanted cell-seeded scaffolds. Enhanced vascularization of the silk fibroin scaffolds has been achieved by integrating a mixture of endothelial cells with osteoblasts (Unger et al., 2010), but also by adding only one of these two cell populations (Unger et al., 2010; Ghanaati et al., 2011). Here we analyzed the capacity of human dental pulp stem cells (hDPSCs) and gingival fibroblasts (hGFs) seeded into silk fibroin scaffolds to attract vessels from the chicken embryo chorioallantoic membrane (CAM). Vascularization of the scaffolds was assessed with magnetic resonance imaging (MRI) and histomorphometric analyses demonstrating that both hDPSCs and hGFs have an equal capacity in attracting vessels within silk fibroin scaffolds. Small discrepancies between MRI and histomorphometric analysis data can be explained by the sample size that was evaluated: histomorphometry assesses only a part of the scaffold, whereas MRI measures the entire scaffold. The high perfusion capacity and the homogenous vessel distribution within hDPSC- or hGF-seeded silk fibroin scaffolds suggest an improved and faster regenerative outcome. The present results also indicate that a relatively long pre-incubation period (i.e., 1 week) of these cells is necessary for obtaining the homogeneous and abundant vascularization of the entire scaffold that favors its integration into the host tissue. Indeed, our previous studies based exclusively on MRI measurements have shown that a shorter pre-incubation period (i.e., 1 day) of hDPSCs is not leading to such a uniform distribution of vessels within silk scaffolds (Kivrak Pfiffner et al., 2015). Comparison between hDPSCs and hGFs seeded scaffolds did not result in obvious differences concerning the attraction of vessels. Similarly to the hDPSCs, hGFs are derived from cranial neural crest cells that possess stem cell properties (Xu et al., 2013; Chiquet et al., 2015; Mitsiadis et al., 2015) and exhibit a higher regenerative potential when compared to fibroblasts of non-oral origin (Eslami et al., 2009) that may explain the similarities observed in vessel attraction into the scaffolds by hDPSCs and hGFs. Previous studies based on gene expression analysis have compared the angiogenic properties of hDPSCs and hGFs and suggested that hDPSCs possess a stronger angiogenic potential (Hilkens et al., 2014). However, studies realized on the CAM have shown that the number of vessels growing into hDPSC- and hGF-containing Matrigel™ droplets was not variable (Hilkens et al., 2014). Vascular ingrowths have been observed into cell-free silk scaffolds after subcutaneous transplantation in mice, but these structures were mainly localized on the scaffold surface (Unger et al., 2010; Ghanaati et al., 2011).

Taken together the present findings clearly demonstrate that hDPSCs and hGFs possess equal capabilities in attracting vessels within silk fibroin scaffolds. Furthermore, these results show that the prolonged pre-incubation period of these two cell populations before their implantation, favors the homogeneous distribution of vessels within silk fibroin scaffolds, a process that guarantees successful tissue regeneration.

## Author contributions

AW: experimental design, performance of experiments, writing of the manuscript, editing, discussing; JB: experimental design, MRI-measurements and analysis, writing of the manuscript, editing, discussing; CW: MRI-measurements and analysis, writing of the manuscript, editing, discussing; BS: experimental design, writing of the manuscript, editing, discussing; TM: experimental design, writing of the manuscript, editing, discussing.

## Funding

This work was supported by the Swiss National Foundation (SNSF) grant 31003A_135633 (TM, AW), by institutional funds from University of Zurich (TM) and by the Matching Funds from the University of Zurich (JB, CW).

### Conflict of interest statement

The authors declare that the research was conducted in the absence of any commercial or financial relationships that could be construed as a potential conflict of interest.
